# Structome: a tool for the rapid assembly of datasets for structural phylogenetics

**DOI:** 10.1093/bioadv/vbad134

**Published:** 2023-10-03

**Authors:** Ashar J Malik, Desiree Langer, Chandra S Verma, Anthony M Poole, Jane R Allison

**Affiliations:** Bioinformatics Institute, Agency for Science, Technology and Research (A*STAR), 138671 Singapore; School of Biological Sciences, University of Auckland, 1142 Auckland, New Zealand; Bioinformatics Institute, Agency for Science, Technology and Research (A*STAR), 138671 Singapore; Department of Biological Sciences, National University of Singapore, 117543 Singapore; School of Biological Sciences, Nanyang Technological University, 637551 Singapore; School of Biological Sciences, University of Auckland, 1142 Auckland, New Zealand; Digital Life Institute, University of Auckland, Auckland 1142, New Zealand; School of Biological Sciences, University of Auckland, 1142 Auckland, New Zealand; Digital Life Institute, University of Auckland, Auckland 1142, New Zealand; Maurice Wilkins Centre for Molecular Biodiscovery, University of Auckland, 1142 Auckland, New Zealand; Biomolecular Interaction Centre, University of Canterbury, 8041 Christchurch, New Zealand

## Abstract

**Summary:**

Protein structures carry signal of common ancestry and can therefore aid in reconstructing their evolutionary histories. To expedite the structure-informed inference process, a web server, Structome, has been developed that allows users to rapidly identify protein structures similar to a query protein and to assemble datasets useful for structure-based phylogenetics. Structome was created by clustering ∼94% of the structures in RCSB PDB using 90% sequence identity and representing each cluster by a centroid structure. Structure similarity between centroid proteins was calculated, and annotations from PDB, SCOP, and CATH were integrated. To illustrate utility, an H3 histone was used as a query, and results show that the protein structures returned by Structome span both sequence and structural diversity of the histone fold. Additionally, the pre-computed nexus-formatted distance matrix, provided by Structome, enables analysis of evolutionary relationships between proteins not identifiable using searches based on sequence similarity alone. Our results demonstrate that, beginning with a single structure, Structome can be used to rapidly generate a dataset of structural neighbours and allows deep evolutionary history of proteins to be studied.

**Availability and Implementation:**

Structome is available at: https://structome.bii.a-star.edu.sg.

## 1 Introduction

The determination of protein structures has become more routine over the last two decades leading to a substantial growth of data in public repositories [e.g. RCSB Protein Data Bank (PDB), rscb.org]. In addition, the step-change in accuracy and accessibility of protein structure predictions yielded by the development of AlphaFold2 (Jumper *et al.* 2021) and RosettaFold (Baek *et al.* 2021), along with the huge scale of the AlphaFold protein structure database (Varadi *et al.* 2022) has enormously increased the number and nature of protein structures available. Furthermore, the ability to study the dynamical behaviour of proteins may yield insights into function (Porebski *et al.* 2012, Sutter *et al.* 2015, Lee *et al.* 2019, Allison 2020) and aid in structure-informed drug design (Christophe *et al.* 1994, Kuhn *et al.* 2002, Martin *et al.* 2017, Śledź and Caflisch 2018).

The ready availability of protein structures has proven valuable for function determination (Li *et al.* 2018) and evolutionary analysis of proteins (Caetano-Anollés and Caetano-Anollés 2003, Yang *et al.* 2005, Brindefalk *et al.* 2013, Krupovic and Koonin 2017, Longo *et al.* 2020, 2022), with the structure-similarity-based classification databases SCOP (Fox *et al.* 2014) and CATH (Dawson *et al.* 2017) providing invaluable support in both aspects. However, for novel protein structures or those not characterized by the aforementioned databases, characterization through determination of structure-based similarity remains an important step, which is further exemplified by the availability of a vast number of protein structure comparison tools e.g. TM-Align (Pandit and Skolnick 2008), MAMMOTH (Ortiz *et al.* 2002), CE (Shindyalov and Bourne 1998), FATCAT (Veeramalai *et al.* 2008), Dali (Holm and Park 2000), secondary-structure matching (SSM) (Krissinel and Henrick 2004), and Foldseek (van Kempen *et al.* 2022).

**Figure 1.**
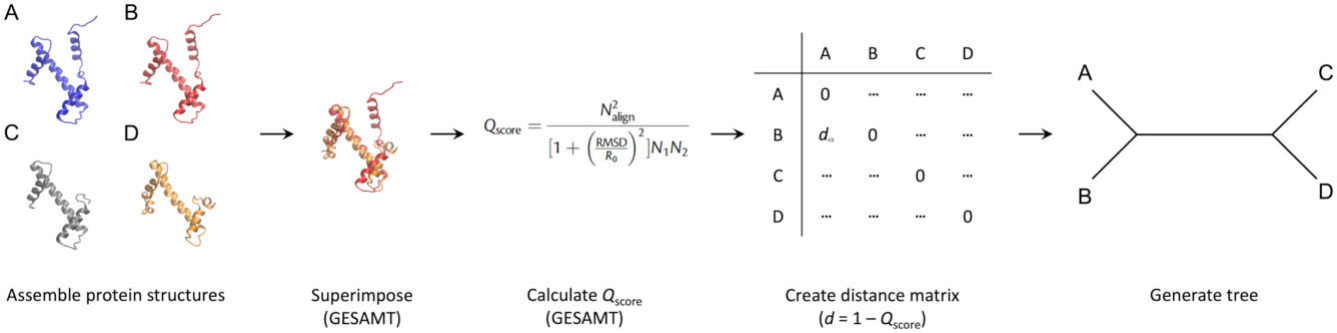
Overview of structural phylogenetics. Protein structures can be obtained from online databases, such as the RCSB PDB. They should be checked for completeness and trimmed or filled as necessary. The software package GESAMT (Krissinel 2012) carries out pairwise structure-based superpositions by aligning vectors representing secondary-structure elements and calculates the $Q_{\mathrm{score}}$ quantifying the quality of the superposition, which depends on the number of amino acid residues in each protein structure that can be superimposed ($N_{1}$, $N_{2}$), the Cα RMSD of these residues, and a scaling factor ($R_{0}$). The $Q_{\mathrm{score}}$ values are converted to distances ($1-Q_{\mathrm{score}}$) and assembled into a distance matrix, which can be used to build a phylogenetic tree showing the evolutionary relationships between the protein structures.

Structural data are increasingly being used to assess evolutionary relationships between proteins. In conventional sequence-based phylogenetics, these relationships are explored through identifying similarities between protein sequences. However, sequence changes more than structure on equivalent time-scales (Illergård *et al.* 2009), meaning that deep evolutionary signals may be missed if comparisons are based on sequence alone. Structure-based phylogenetics (or structural phylogenetics, see Fig. 1 for an overview) leverages the robustness of protein structures to change on evolutionary time-scales to detect and quantify deep evolutionary relationships in cases where sequence similarity is limited (Breitling and Gerber 2000, Bujnicki 2000, Garau *et al.* 2005, Abrescia *et al.* 2012, Lundin *et al.* 2012, 2015, Malik 2018, Malik *et al.* 2020).

To aid in the rapid exploration of protein evolution from structure, we have developed Structome, a web-based resource for identifying structure-based phylogenetic relationships. Using Structome, we show that it is possible to rapidly generate a structural phylogeny starting with a single structure as input. We illustrate this capability by producing a structural phylogeny to analyse the evolutionary history of the histone fold (Alva *et al.* 2007). It is possible to generate detailed sequence-based phylogenies for individual histones (Thatcher and Gorovsky 1994), but use of structure allows a phylogeny to be built that unites all proteins sharing this common fold.

## 2 Methods

All data handling was carried out using the bash shell and Python v3.8.8, with numpy v1.20.1. Python was managed using conda environments.

### 2.1 The Structome database

**Figure 2.**
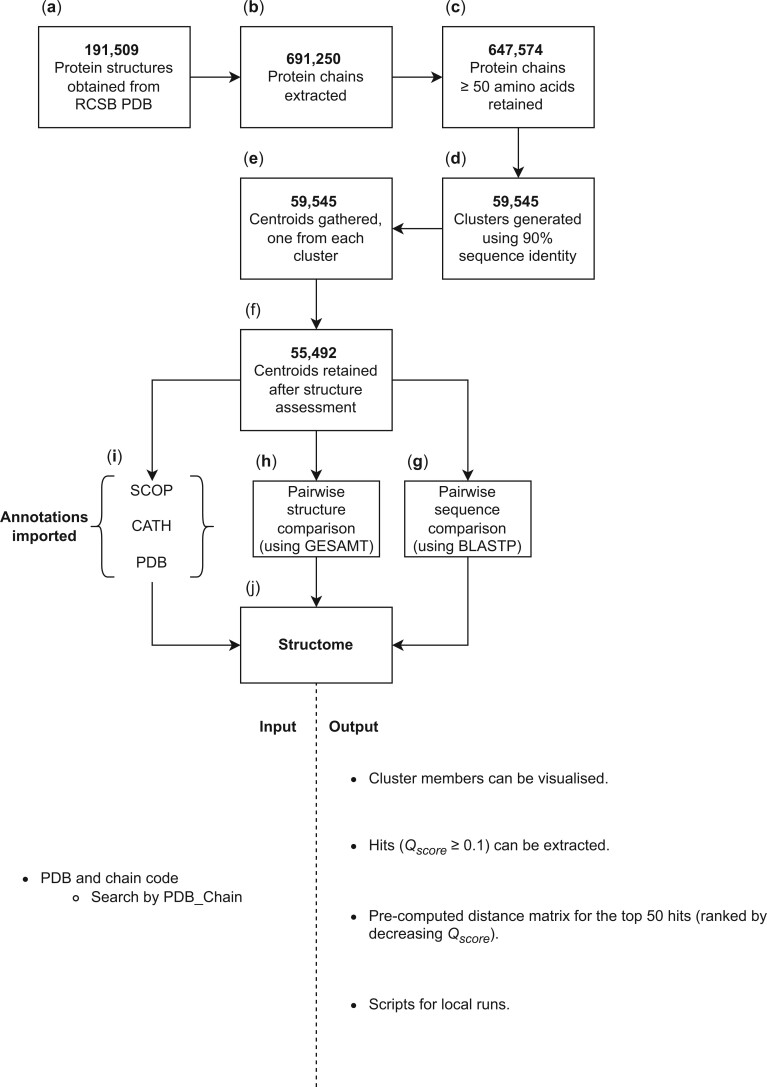
The Structome database, queries and responses. Protein structures were acquired from RCSB PDB (a), split into individual chains (b) and filtered on protein sequence length, with those longer than 50 amino acids retained (c). The proteins were clustered at 90% sequence identity (d) and cluster centroid structures chosen as representatives of each cluster (e and f). Protein centroids were then pairwise compared to get protein structure similarity and protein sequence similarity (g and h). These data, together with annotations from SCOP, CATH, and the PDB, are included in Structome. The bottom part of the diagram shows how to provide input to Structome using the query format PDB_Chain. The Structome output allows the user to visualize the structures of all members of each cluster superimposed on the cluster centroid, extract centroids with $Q_{\mathrm{score}}\geq 0.1$ compared to the query cluster centroid, and obtain a pre-computed distance matrix in nexus format for the 50 centroids most similar to the query cluster centroid. It is also possible to download scripts to generate distance data locally.

Protein structures and sequences were acquired from the RCSB PDB. Each protein was decomposed into its constituent chains using VMD (Humphrey *et al.* 1996), followed by the implementation of a protein size (amino acid count in protein sequence) cut-off. The remaining proteins were clustered at 90% sequence identity using usearch (Edgar 2010) and each cluster was represented by its centroid. Each centroid was assessed and removed from Structome if it did not meet certain criteria, explained in more detail below. The final dataset of all centroid structures were pairwise compared using General Efficient Structural Alignment of Macromolecular Targets (GESAMT) (Krissinel 2012) and BLASTP to generate protein structure and sequence comparison statistics. These data, along with annotations imported from the PDB, SCOP, and CATH, are provided in the Structome database. A step-by-step illustration of the process outlined above is illustrated in Fig. 2, and more detail surrounding each of these steps is provided below. The current version of Structome was built from the complete set of structures in the PDB downloaded on 22 September 2022. Structome is planned to be updated annually in the future.

### 2.2 Determination of protein size

The protein size used for the size-based filtering of protein chains during the process of building Structome (see Fig. 2c, protein chains retained) was the number of amino acids in the author-deposited fasta-format sequence record. This was enumerated for each protein chain in the RCSB PDB, and only those comprising 50 or more amino acids were kept for further analysis.

All protein size information reported in Structome, e.g. ‘Sequence length’, refers to the number of amino acids in the complete sequence of the protein chain and not the number of amino acids for which there are structural data.

### 2.3 Protein similarity filtering

Protein sequences were clustered with a cut-off of 90% sequence identity using usearch (Edgar 2010). The structure corresponding to the sequence of the centroid of each cluster was considered a representative of all the members of that cluster. The clustering step helped remove redundancy caused by (i) multiple structures for the same protein, (ii) homo-multimeric proteins, and (iii) structures whose sequences differ only by point mutations.

Given the automated nature of the clustering algorithm, expecting artefacts, a binary measure of confidence was associated to each of the members of a cluster. Given the semi-global alignment-based clustering, it was expected that all members of a cluster have the same size. Members with sizes more than 1 SD away from the cluster median size were classified as having low confidence.

### 2.4 Protein structure comparison

SSM (Krissinel and Henrick 2004) was used by us previously (Malik 2018, Malik *et al.* 2020) to compare protein structures. It computes $Q_{\mathrm{score}}$, a measure of structural similarity that is attractive as it includes both alignment quality and length. To build Structome, an improved version of the same program, GESAMT (Krissinel 2012) provided as the program gesamt in the Collaborative Computational Project No. 4 suite of programs (Collaborative Computational Project, Number 4 1994) was used to compare protein structures. Protein structures that were provided by RCSB PDB in non-standard formats were discarded.

Protein structure comparison, as with sequence comparison, was carried out in two steps (see Fig. 2f and h, centroid structure assessment and pairwise structure comparison). In the first step, the centroid structures identified using protein sequence clustering were compared with themselves to check whether the GESAMT program was able to analyse its secondary-structure elements. The choice of only including proteins with 50 amino acids or more aimed to ensure that sufficient secondary structure is present for GESAMT to work with.

If GESAMT failed to compare a protein structure with itself, another member of the cluster was chosen and subjected to the same test. This process was repeated until a suitable centroid was identified, which then replaced the original centroid. In case of a singleton cluster or if an alternative centroid could not be selected, the cluster was removed from Structome.

In the second step of protein structure comparison, the centroid structures were compared with one another using GESAMT to obtain the superposition quality score, $Q_{\mathrm{score}}$, which measures their degree of structural similarity. $Q_{\mathrm{score}}$ values range from 0 to 1; a $Q_{\mathrm{score}}$ value of 1 indicates identical structures. If the structural comparison was unable to find any structural similarity it does not report a value of zero, but instead displays a message indicating that no similarity could be detected between shared structures. In Structome, a value of ‘−1’ was assigned to such cases for ease of processing numeric data; however, this value is subsequently replaced with zero in order to compute a distance value. A distance measure can be obtained from the $Q_{\mathrm{score}}$ according to $1-Q_{\mathrm{score}}$. For each centroid structure, a matrix containing the distance measures for the 50 centroid structures with the highest $Q_{\mathrm{score}}$ values is provided in the nexus-formatted output file that can be downloaded from Structome. Only the top 50 centroid structures are considered here for reasons of computational tractability.

### 2.5 Protein sequence comparison

A BLAST protein database comprising the centroid sequences was generated using BLAST v2.8.1+ (Altschul *et al.* 1990). Each centroid sequence was then compared to the database to provide information about sequence similarity and identity. For each comparison, the word size for BLASTP hits was changed to two amino acids to ensure a score was generated for most comparisons, accompanied by an *E*-value. A score of ‘−1’ was assigned to all centroid pairs where BLASTP could not find similarity across the proteins compared. The sequence similarity and identity data are provided in Structome.

### 2.6 SCOP and CATH classifications

For each protein structure centroid used in Structome, SCOP (Murzin *et al.* 1995) and CATH (Orengo *et al.* 1997) classifications were obtained, where available. Missing classifications are reflected by ‘N/A’ in Structome.

### 2.7 Web deployment

Structome is designed as a web app making use of the open-source Python library, Streamlit. All navigation within web pages uses only in-app hyperlinks. Protein structure visualization in Structome uses py3Dmol, a python wrapper for the 3Dmol javascript library (Rego and Koes 2015).

### 2.8 Illustration of usage: histone fold

Structome was queried using the histone H3 fold PDB ID 4uuz, chain A. The sequence of the centroid of the Structome cluster to which this protein belongs, PDB ID 1p3m, chain A, was compared to that of PDB ID 4uuz, chain A, using BLASTP, and their structures compared using GESAMT. The transformation matrix generated by GESAMT was used to superimpose the two structures using VMD.

All protein structures in the same Pfam (Mistry *et al.* 2021) family (PF00125) in which centroid protein PDB ID 1p3m, chain A is classified were downloaded using the InterPro (Paysan-Lafosse *et al.* 2023) API.

The sequence of centroid structure PDB ID 1p3m, chain A, was submitted to the HHPred online server (Zimmermann *et al.* 2018, Gabler *et al.* 2020) (link: https://toolkit.tuebingen.mpg.de/tools/hhpred) and hhsearch was used to identify protein structures with which it shares remote homology.

## 3 Results

The motivation behind Structome was to develop a resource which, given a query protein structure, can rapidly show the neighbouring protein structures based on structural similarity and, additionally, use this neighbourhood to populate a dataset that can be used to explore structure-based phylogenetic relationships. Figure 2 shows a step-by-step breakdown of the creation of the Structome database. This, together with the web interface and what the user may achieve, is described below.

### 3.1 Protein structure selection

All 191 509 PDB files (as on 22 September 2022) were downloaded from RCSB PDB. At the current stage of development of Structome, only experimentally determined structures are included. As our understanding of low quality regions in predicted structures, e.g. those predicted by AlphaFold2 (Jumper *et al.* 2021, Varadi *et al.* 2022) or ESMFold (Lin *et al.* 2022), improves, future releases of Structome will see these structures included in this resource.

Each PDB file was split into monomers (protein chains), with each of the 691 250 chains considered an individual protein structure. Hereafter, ‘protein’ refers to an individual protein chain. Given the breadth of structures deposited in RCSB PDB, each protein chain could comprise a small peptide (protein sequence length ≤50 amino acids), a single domain or a multi-domain protein. For instance, a number of protein structures in the RCSB PDB database are short peptides (e.g. chain B, from the PDB structure 7jls, which is a tri-peptide, and chain P from PDB structure 5ntn, which is only a 20 amino acid long segment of a larger protein). On the other hand, the solute carrier family protein (PDB ID 6y5v, chain A) contains two domains at a structural level (Supplementary Fig. S2), although these are often treated as three domains at a functional level (Chi *et al.* 2021).

To ensure that the structures considered had sufficient regular secondary-structure content and sufficiently long sequences for meaningful structure- and sequence-based comparisons, an empirical size threshold of 50 amino acids was used, with only proteins of 50 amino acids or more included in the Structome dataset. This size-based exclusion still retained 647 574 protein structures (i.e. ∼94% of protein structures in RCSB PDB).

### 3.2 Protein clustering

Several entries in the RCSB PDB comprise structures with highly similar or identical sequences. For example, some proteins are represented by a large number of structures, especially those that are commonly used to validate structure determination methods. Others are homo-multimers where each chain has an identical sequence and near identical structure, e.g. the crystal structure, PDB ID 1hv4, contains the deoxy form of haemoglobin from *Anser indicus*, which is tetrameric, comprising two α-haemoglobin chains and two β-haemoglobin chains. In other instances, structures of the same protein with single or multiple mutations are present, e.g. in the case of tumour suppressor protein p53, several mutated variants (e.g. PDB IDs: 4kvp, 4loe, 4lof, and 4lo9) are present with negligible change in protein structure.

To remove the resulting redundancy in the set of structures, following the size-based exclusion, the protein sequences were clustered with a cut-off of 90% sequence identity using usearch. The usearch program identified 59 546 clusters based on the sequence identity cut-off. The centroid of each cluster was considered to be representative of the structures in that cluster, and subsequent sequence and structure comparison was carried out only for these centroids. This greatly reduced the redundancy in the protein structure dataset while simultaneously making the subsequent analysis computationally tractable.

The five largest clusters contain 3631, 1602, 1426, 1413, and 1304 protein structures, respectively, with their centroids being a viral capsid protein (PDB ID 2gon, chain A), β-2 microglobulin (PDB ID 1ypz, chain B), a chemically synthesized ubiquitin (PDB ID 1yiw, chain A), a viral spike glycoprotein (PDB ID 7eya, chain N), and actin (PDB ID 1d4x, chain A). The reason for the presence of the capsid protein in the top five is due to the presence of complete viral capsid structures, such as PDB ID 3j3q and 3j3y, which have 1356 and 1176 units of the HIV capsid protein, respectively. Actin also has several multimeric assemblies in the RCSB PDB. The remaining three centroids are either model systems frequently used for testing structure determination methods (e.g. ubiquitin) or have important immunological roles (e.g. the viral spike glycoprotein), which explains their popularity as systems of interest. A large proportion of the clusters, 42%, have only one or two structures, and a further 22.53% have between three and five structures (Table 1), showing that there are many protein structures in the RCSB PDB that may be considered to be at least somewhat unrelated to other entries on a sequence level. The entire list of clusters and their members can be found in the Supplementary Material (Supplementary Table S1).

**Table 1.** Proportion of the 55 492 clusters that contain the specified numbers of proteins.

Clusters (%)	Number of member proteins
21.45	1
20.55	2
22.53	Between 3 and 5
22.63	Between 6 and 15
9.58	Between 16 and 50
3.26	> 50

### 3.3 Protein comparison

Protein structural similarity was calculated for all pairs of cluster centroids using GESAMT, returning a $Q_{\mathrm{score}}$ value for each pairwise comparison. In the first instance, self-comparisons were used to identify cluster centroids whose structure was incomprehensible to GESAMT. These cluster centroids were replaced with another member of the cluster (see Section 2). This process was repeated until either a suitable replacement was found, or there were no structures in the cluster able to be processed; in the latter case, that cluster was removed from the dataset. This screening process reduced the number of clusters to 55 492.

**Figure 3.**
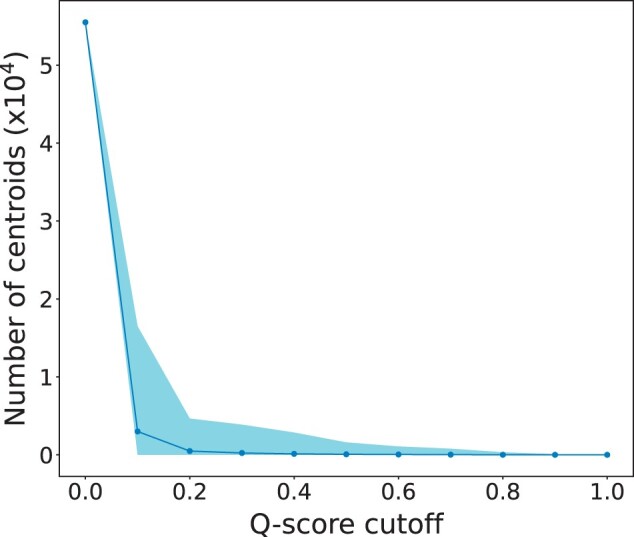
The number of centroid pairs with $Q_{\mathrm{score}}$ values greater than a given $Q_{\mathrm{score}}$ cut-off increases rapidly as the cut-off drops. The solid line represents the average number of pairs that score better than a given cut-off. The shaded region represents the amount of variation at each cut-off, for the 55 492 centroids.

The remaining centroid protein structures were pairwise compared, returning a $Q_{\mathrm{score}}$ value for each comparison. Values approaching zero indicate low structural similarity, and a value of one indicates that the two structures are identical. The number of structures with $Q_{\mathrm{score}}$ values greater than a given cut-off is illustrated in Fig. 3. The number of pairwise comparison scores increases exponentially as the $Q_{\mathrm{score}}$ cut-off decreases. This is equivalent to the increased noise in sequence-based analysis observed by Rost (1999) when approaching the so-called ‘twilight zone’ of sequence similarity; a region of the sequence similarity versus alignment length landscape, where it becomes increasingly difficult to separate between protein pairs of similar and non-similar structure. $Q_{\mathrm{score}}$ values below 0.1 may therefore be considered as the structural analogy of the twilight zone. Consequently, Structome only provides comparison results that generate $Q_{\mathrm{score}}$ values ≥0.1.

In some cases, even a $Q_{\mathrm{score}}$ cut-off of 0.1 may be too generous. For instance, despite size differences contributing to $Q_{\mathrm{score}}$, querying Structome with the solute carrier family protein 6y5v chain A produces apparent hits with $Q_{\mathrm{score}}>1$ for which only one of the two structural domains is present and that upon closer scrutiny is clearly not homologous and cannot be used in phylogenetic inference (Supplementary Fig. S2). While Structome provides a convenient interface for generating datasets with which to investigate evolutionary relationships, the data still require careful inspection.

For the purpose of phylogenetic inference, which we see as a key end usage of the structural datasets identifiable using Structome, a distance rather than a similarity measure is required. Following our previous work developing methodology for carrying out structure-based phylogenetics (Lundin *et al.* 2012, Malik *et al.* 2020), the $Q_{\mathrm{score}}$ values are converted into a distance measure according to $1-Q_{\mathrm{score}}$. It is this distance measure that is used to generate the neighbour-joining trees displayed on Structome and in the distance matrices that can be exported from Structome.

The sequences of each pair of centroid proteins were also compared using BLASTP to complement the structure comparison and provide an insight into any relationship between sequence and structure.

### 3.4 Structome

The Structome web server provides users with a graphical web interface from which to search, organize, and visualize the structure- and sequence-based data described above. It also allows export of the structural similarity data in tabular form, and as a nexus-formatted distance matrix accompanied by a neighbour-joining tree. Below, the content and functionality of Structome is described and then illustrated with an example.

Users begin their interaction with this resource at its index page (base url: http://structome.bii.a-star.edu.sg/). The index page presents four navigational hyperlinks, namely (i) ‘Home’; to navigate to the index page from any point in Structome, (ii) ‘Explore neighbours’; to look for other proteins similar in structure to the user-provided protein structure, (iii) ‘Explore clusters’; to see which usearch-defined cluster a user-submitted protein belongs to, and (iv) ‘Run locally’; to navigate to a repository comprising some information about the content of Structome and to download a set of python scripts to generate comparison data and the associated neighbour-joining tree.

### 3.5 Explore neighbours

The ‘Explore neighbours’ hyperlink navigates to a page where the user can input the PDB ID and chain ID of their protein chain of interest to the ‘PDB_Chain’ text input field (e.g. 1p3m_A or 1hv4_A). Submitting a suitable ‘PDB_Chain’ combination will result in (i) a text statement explaining the contents of the page, (ii) a neighbour-joining phylogenetic tree, and (iii) a table of results. There are also two buttons; ‘Download table data as CSV’ allows users to download the table of results ranked by $Q_{\mathrm{score}}$, and ‘Download tree data’ allows users to download a distance matrix of the top-50 hits by $Q_{\mathrm{score}}$ and the neighbour-joining tree displayed in Structome. Each row of the results table displays the results of the comparison between the centroid of the cluster to which the user-submitted structure belongs and another centroid. The rows are interactive and can be clicked by the user to access a superposition of that pair of centroid structures (which can be downloaded by clicking the ‘Download aligned structures’ button) and detailed data regarding their structure and sequence comparison. Overall, the ‘Explore neighbours’ function allows users to examine the proteins that are structurally similar to their query protein and, if desired, download these data for further analysis.

### 3.6 Explore clusters

The ‘Explore clusters’ hyperlink also navigates to a page where the user can input the PDB ID and chain ID of their protein chain of interest to the ‘PDB_Chain’ text input field (e.g. 1p3m_A or 1hv4_A). However, in this case, submitting a query ‘PDB_Chain’ will return a table listing the members of the cluster to which the query belongs. It is the centroid of this cluster that is used for the pairwise comparisons whose results are displayed when using the ‘Explore neighbours’ function. The rows in the table on the ‘Explore clusters’ page are also interactive and a click on a row will result in display of the superposition of that member of the cluster and the cluster centroid. Overall, the ‘Explore clusters’ function allows users to see how similar their query structure is to the centroid of its cluster and, more generally, to explore the structural variation within a cluster.

### 3.7 Data visualization

The tables provided in Structome contain useful structural similarity data, but are not an intuitive way to understand the structural neighbourhood of a cluster centroid. Visualization as a phylogenetic tree or network, either on the Structome web server or generated locally from exported distance matrix data is a better way to achieve this. To assist with visualization, and to allow users to include protein structures not included in Structome, we have provided opportunities for the data to be downloaded (from the ‘Explore neighbours’ page, see Section 3.5) as well as scripts for custom local analysis (obtained using the ‘Run locally’ hyperlink).

### 3.8 Illustration of usage: a structural phylogeny of histone fold-containing proteins

To illustrate the utility of Structome in rapidly assembling datasets for structural phylogenetics, we walk through the steps depicted in Fig. 1 for the histone fold. This is an interesting test case because it is recognized as being an ancient protein fold with a deep and complex evolutionary history (Alva *et al.* 2007). The identification of histone-fold proteins thus involves careful structural comparison and, moreover, a united sequence-based phylogeny is not practical; sequence-based phylogenies are thus restricted to individual histones (Thatcher and Gorovsky 1994)

**Figure 4.**
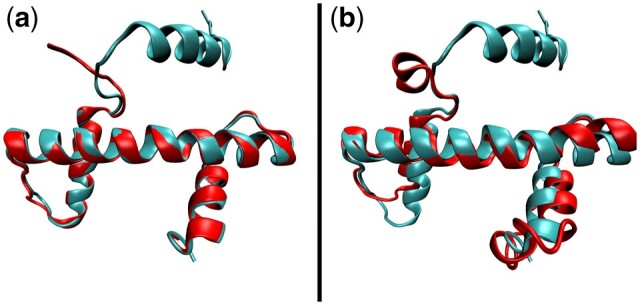
Histone-fold superposition. (a) Superposition of the structures of PDB ID 4uuz, chain A (red) with the centroid of the cluster to which it belongs (cluster ID 01052), PDB ID 1p3m, chain A (cyan). Of their 136 and 135 amino acids, respectively, 76 can be superimposed. (b) Superposition of the structures of the centroid of cluster 08189, PDB ID 6m4g, chain C (red), and the centroid of cluster 01052, PDB ID 1p3m, chain A (cyan). The $Q_{\mathrm{score}}$ for comparison of these structures is 0.409, and 72 of their 115 and 135 amino acids, respectively, can be structurally aligned; this covers the conserved histone fold (Alva *et al.* 2007).

As per Fig. 1, we first assemble a dataset. To do this, we arbitrarily chose the chain corresponding to histone H3 (chain A) from PDB ID 4uuz, which derives from *Drosophila melanogaster* (Richet *et al.* 2015). Querying Structome via the ‘Explore neighbours’ tab reveals that 4uuz_A is a member of a cluster for which the centroid structure is PDB ID 1p3m chain A (1p3m_A; histone H3 from *Xenopus laevis*). The two proteins share 97% sequence identity, and have nearly identical core structures (Fig. 4a), so the centroid is very similar to the initial query structure. This justifies the clustering of proteins with highly similar sequences and use of the resulting cluster centroids to represent all proteins in a given cluster in Structome.

**Figure 5.**
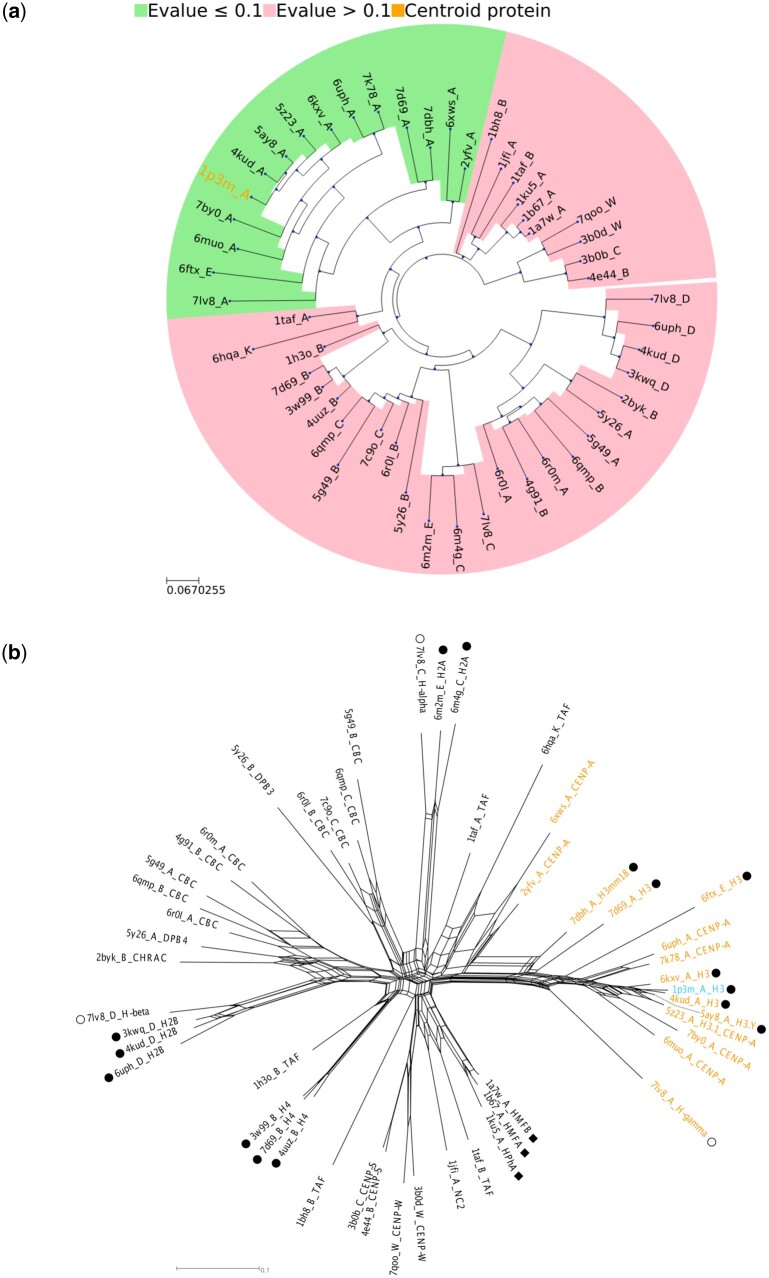
Histone-fold structural phylogenies of the 50 cluster centroids most structurally similar to centroid 1p3m_A (*X.laevis* histone H3), the centroid of the cluster to which the query protein (4uuz_A) belongs. (a) Histone-fold phylogeny in the format automatically produced by Structome, coloured by BLASTP *E*-value as indicated. Each label contains the PDB ID and chain of each cluster centroid, and the query centroid is in orange. (b) A neighbornet network of the histone-fold phylogeny. The query centroid (1p3m_A) is in cyan. Each label contains the PDB ID and chain code followed by a classification obtained from the RCSB PDB or associated literature: H2A, H2B, H3, H4-core histone proteins (solid circles); HMFA, HMFB, HPhA-archaeal histones (diamonds); H-alpha, H-beta, H-gamma-viral histones (hollow circles); TAFF-TATA-Associated Factors; CBC-CCAAT-Box Binding Complex; DBP3, DPB4-DNA Polymerase Binding Protein 3, 4; CHRAC-Chromatin Accessibility Complex; CENP-A, CENP-S, CENP-W-Centromere Protein A, S, W; NC2-Transcription regulator NC2. A comprehensive list of centroids and the taxa from which they derive is available in the Supplementary Material. Departures from tree-likeness in the network indicate the existence of alternative interpretations of the data. The distance matrix was downloaded from Structome and the network was created using SplitsTree (Huson and Bryant 2006), with the compressed protein descriptor and partitions added during post-processing to make the network easily interpretable. The taxa in orange indicate those that are recoverable by BLASTP-based sequence similarity search resulting in *E*-values below 0.1. The remaining taxa either have very high *E*-values or are not detectable as hits by BLASTP.

The results from using Structome to explore neighbours of PDB ID 4uuz chain A (represented by cluster centroid 1p3m_A) include a table of hits to other centroids (5358 centroids with $Q_{\mathrm{score}}>0.1$ when compared to 1p3m_A), plus an automatically generated phylogeny derived from distance data (Fig. 5a), which highlights the centroid 1p3m_A (orange). This phylogeny is unrooted; the apparent root node does not represent an evolutionary interpretation but is simply an artefact of the ete3 tree-drawing library. The tree is subdivided based on *E*-value (from BLASTP). This serves to indicate to the user the part of the structural dataset that is likely to be readily identified by sequence similarity from the part of the structural dataset that may be more challenging to identify on the basis of sequence data alone (*E*-value >0.1). As an example, the results table reveals that among the top 50 hits, 6m4g_C (a subtype of histone-2A, H2A from *Homo sapiens*) has 39% sequence similarity to the centroid 1p3m_A but a high *E*-value of 423 327.

The $Q_{\mathrm{score}}$ for this comparison is 0.409, with 72 residues structurally aligned (Fig. 4 and Supplementary Fig. S1). These superimposable residues cover the conserved histone fold (Alva *et al.* 2007). Thus, Structome returns proteins that have clear and meaningful structural similarity that are unlikely to be detected with standard usage of BLASTP.

To better assess the resulting tree, we present a structural phylogenetic network (Lundin *et al.* 2012, Malik *et al.* 2020) created from the pre-calculated distance matrix for the 50 centroids most structurally similar to centroid 1p3m_A that can be downloaded from Structome (Fig. 5b). This illustrates that the data are tree-like (the more the network resembles a tree, the fewer conflicts there are in the data) and that key protein groups are recovered as clans (possible monophyletic groups in an unrooted tree). In brief, each of the four major eukaryotic histones (H2A, H2B, H3, and H4) forms a distinct clan. This shows that the network is consistent with the monophyletic origin of each of these histones.

As we started with the structure of histone H3 (4uuz_A), we can see a bias towards H3 structures, which are better represented than the other major histone proteins. This is not surprising as any search will rank hits by similarity. This serves as an indicator that, for a careful assessment of structural evolutionary relationships, one may wish to run more than one search in the data collection phase, as the query structure may bias the datasets collected.

It is not clear from the tree exactly how each eukaryotic histone may have evolved from a shared common ancestor, but it is interesting that each appears to have non-histone neighbours. This could be an indicator of a complex evolutionary history (multiple independent recruitments of the histone fold into the nucleosome). For example, the relationship between H2B and a group of TAFs including CCAAT-Binding Complex proteins shows a clear split, which is suggestive of a common evolutionary origin (though we have not examined whether these TAFs have evolved from H2B or vice versa).

Alternatively, it is possible that the core histones (solid circles) are very ancient and their divergence from a shared common ancestor is obscured. One clear result suggests that care is warranted in interpreting this phylogeny. We see a clan that groups the archaeal histones (diamonds) together, but the nearest neighbours are centrosomal proteins from vertebrates. These proteins are known to be fast evolving (Malik and Henikoff 2003), and in all probability are much younger than the core eukaryotic histones. This association may therefore be the result of different rates of evolution dragging these derived vertebrate structures close to very ancient proteins (archaeal histones)—a structural equivalent of a long-branch attraction artefact.

Finally, it is noteworthy that the viral histones (hollow circles), which are represented here by structures from the giant Marseillesvirus, each appear to be closely related to core eukaryotic histones. This relationship fits the expectation that such viruses are related to eukaryotes and not a fourth domain of life (Moreira and Brochier-Armanet 2008, Yutin *et al.* 2014).

### 3.9 Comparison with Pfam and HHPred

The structural neighbourhood of the histone-fold centroid structure used to illustrate Structome usage above, the H3 histone 1p3m_A, was also investigated using Pfam and HHPred. Pfam groups proteins that are homologous into families, using profile hidden Markov models, which are more sensitive than conventional BLASTP analysis, and HHPred is a powerful method for detecting remote sequence homology.

The centroid 1p3m_A is classified under the Pfam family ‘Core histone H2A/H2B/H3/H4’ (Pfam ID PF00125). The Pfam Core histone H2A/H2B/H3/H4 family comprises 955 proteins, which are separated into 3971 protein chains (monomers). Of these 3971 proteins chains, 643 are not present in Structome because of size-based exclusion (e.g. 6bhd_B comprises 18 amino acids, 4i51_C comprises 9 amino acids, etc., which are below the 50 amino acid cut-off for inclusion in Structome).

Interestingly, 30 structures in the Pfam Core histone H2A/H2B/H3/H4 family returned a $Q_{\mathrm{score}}$ value <0.1, and hence were not considered as hits in Structome due to low apparent structural similarity. These 30 structures can generally be divided into two categories: (i) the structure is in a cluster in which the centroid does not have the histone fold (e.g. 6nzo_C, labelled as Ubiquitin-60S ribosomal protein L40, Histone H2A, is in the cluster represented by centroid 1yiw_A, a chemically synthesized ubiquitin) or (ii) the structure does present a histone fold, but the proteins being compared have a significant size mismatch leading to a bias in $Q_{\mathrm{score}}$ calculations resulting in low $Q_{\mathrm{score}}$ values, an effect that has been explored in detail elsewhere (Malik *et al.* 2020). The remaining 3298 proteins in the Pfam family map to 52 of the 5358 clusters returned as hits for the centroid 1p3m_A.

On the flip side, Structome identifies histone-like folds that are not in the Pfam family ‘Core histone H2A/H2B/H3/H4’, which groups nucleosome histones and does not exhaustively include all histones. For example, 6m4g_C is classified under a different Pfam family (Pfam ID P0C5Z0, Histone H2A-Bbd type 2/3). Structome can therefore be of use in gathering a broader set of structurally similar proteins.

Querying HHPred with the sequence of 1p3m_A returned 60 proteins. Of these, 33 have an *E*-value >0.1, as computed by hhsearch, meaning that the sequence comparison is unreliable. Three of the 60 proteins returned $Q_{\mathrm{score}}$ values <0.1 when compared to 1p3m_A and so were not considered hits by Structome. Upon investigation, these low $Q_{\mathrm{score}}$ values are due to size differences. The remaining 57 hhsearch hits were mapped to 44 of the 5358 clusters returned as Structome hits for the centroid 1p3m_A.

The above results demonstrate that both sequence- and structure-based analysis for inferring phylogenetic relationships between (i) histones themselves and (ii) between histones and other DNA-binding proteins are not trivial. Structome provides the ability to use a query structure rather than sequence to populate a dataset of structurally similar proteins that may captures remote evolutionary signals hidden in protein structure. This, coupled with the capability to download and run scripts locally to generate structure-based comparisons for a set of user-defined structures, should greatly facilitate the investigation of deep evolutionary relationships through structure-based phylogenies.

## 4 Conclusion

Structome is a web server that allows users to search for protein structures that are structurally similar to a query protein. First, the centroid of the sequence similarity-based cluster to which the query structure belongs is identified, and the centroid is then used to identify other clusters for which the centroid falls within a user-defined structural similarity threshold. These centroid structures are listed along with additional information, such as SCOP and CATH classifications. A summary page for each cluster allows users to examine other structures in the cluster. The user can export tabular results and a distance matrix for the 50 centroid structures most similar to that of the cluster to which their query structure belongs for further analysis and visualization as a phylogenetic tree or network. Lastly, the user can also export the structures and scripts to run the analysis locally to add value beyond what is provided by the pre-calculated distance data. Structome therefore provides several ways for users to identify and analyse the structural neighbourhood of any given protein structure in the RCSB PDB, with the aim of providing insight into the organization of the protein structure landscape, in particular with respect to the evolutionary history of proteins, through analysis of their structural relationships.

## Supplementary Material

vbad134_Supplementary_Data

## Data Availability

Protein structure data was obtained from RCSB PDB. All protein structure comparison data can be downloaded from Structome: https://structome.bii.a-star.edu.sg/.
